# Blocking Endogenous Leukemia Inhibitory Factor During Placental Development in Mice Leads to Abnormal Placentation and Pregnancy Loss

**DOI:** 10.1038/srep13237

**Published:** 2015-08-14

**Authors:** Amy Winship, Jeanne Correia, Tara Krishnan, Ellen Menkhorst, Carly Cuman, Jian-Guo Zhang, Nicos A. Nicola, Evdokia Dimitriadis

**Affiliations:** 1 MIMR-PHI Institute of Medical Research, 27-31 Wright St, Clayton, VIC, 3168, Australia; 2 Department of Anatomy and Developmental Biology, Wellington Road, Monash University, Clayton, Victoria, 3800, Australia; 3 Faculty of Medicine, Nursing & Health Sciences, Wellington Road, Monash University, Clayton, Victoria, 3800, Australia; 4 The Walter and Eliza Hall Institute of Medical Research, 1G Royal Parade, Parkville, Victoria 3052, Australia; 5 Department of Medical Biology, The University of Melbourne, Parkville, Victoria 3010, Australia

## Abstract

The placenta forms the interface between the maternal and fetal circulation and is critical for the establishment of a healthy pregnancy. Specialized trophoblast cells derived from the embryonic trophectoderm play a pivotal role in the establishment of the placenta. Leukemia inhibitory factor (LIF) is one of the predominant cytokines present in the placenta during early pregnancy. LIF has been shown to regulate trophoblast adhesion and invasion *in vitro*, however its precise role *in vivo* is unknown. We hypothesized that LIF would be required for normal placental development in mice. LIF and LIFRα were immunolocalized to placental trophoblasts and fetal vessels in mouse implantation sites during mid-gestation. Temporally blocking LIF action during specific periods of placental development via intraperitoneal administration of our specific LIFRα antagonist, PEGLA, resulted in abnormal placental trophoblast and vascular morphology and reduced activated STAT3 but not ERK. Numerous genes regulating angiogenesis and oxidative stress were altered in the placenta in response to LIF inhibition. Pregnancy viability was also significantly compromised in PEGLA treated mice. Our data suggest that LIF plays an important role in placentation *in vivo* and the maintenance of healthy pregnancy.

Embryo implantation and subsequent placentation are a continuum involving initially the apposition and adhesion of the blastocyst trophectoderm to the endometrial luminal epithelium^1^. The trophectoderm differentiates into trophoblast cells, which undergo dramatic proliferation, migration and invasion into the endometrium that are critical events ensuring placental development^2^. Equally as important, is the development of the fetal and maternal vasculature, which facilitates the exchange of nutrients, gases and wastes^3^. Inadequate or inappropriate implantation and placentation are major causes of infertility, and are thought to lead to pregnancy loss, placental insufficiency and other obstetric complications^4^. Consequently, placental development is highly regulated spatially and temporally by numerous factors, such as the cytokines produced within the local uterine environment^5^. Such factors can ultimately determine the success or failure of pregnancy.

Leukemia inhibitory factor (LIF) is one of the cytokines predominantly present during early pregnancy. LIF is a member of the interleukin-6 (IL6) family of cytokines and is a secreted glycoprotein that signals via the gp130/LIF receptor (LIFRα) complex to activate downstream signaling cascade including the Janus tyrosine kinase (JAK)/signal transducer and activator of transcription (STAT)^6^,^7^,^8^,^9^,^10^ and extracellular signal-regulated kinase (ERK)^11^ pathways.

LIF has been shown to be indispensible for uterine blastocyst implantation in mice^12^,^13^ and plays a critical role in implantation in primates and women^14^,^15^,^16^. LIF and LIFRα are present in the uterine endometrium and decidua during the peri-implantation stage and early pregnancy in mice^17^. Studies from LIF and LIFR null mice suggest that LIF action on the fetus/placenta is critical for normal development. LIF-null females are infertile due to defects in embryo implantation^15^,^17^, highlighting the role of maternal LIF in the initiation of implantation. However, as implantation fails, these mice are not useful in investigating the role of LIF in placentation. We have shown that blocking endogenous LIF action during the peri-implantation period using a novel polyethylene glycol (PEG) conjugated LIF-antagonist (PEGLA) mimicked this phenotype^13^,^18^.

Conversely, LIFR-knockout mice show complete perinatal lethality, within 24 hours of birth^19^, precluding the generation of adult LIFR^−/−^ mice to investigate the role of LIF in placentation. In these mice, placental morphology is dramatically altered, which likely contributes to the perinatal loss, however given that the fetus is also LIFR-deficient, the precise role of LIF signaling in the placenta *in vivo* remains unknown. These studies do highlight however, the critical importance of LIF action on the fetus/placenta.

LIF and LIFRα have never been localized in the mouse placenta, so it is unclear in which cell types LIF signalling is most important. In women, it is clear that LIF and LIFRα mRNA are expressed in the chorionic villi and decidua of first trimester and term placenta^20^,^21^. *In vitro*, LIF activates STAT3 in human primary extravillous trophoblast (EVT) cells and stimulates their invasion and adhesion to primary endometrial epithelial cells^22^. In trophoblast cell lines, LIF is reported to increase the proliferation and invasion of JEG-3 choriocarcinoma cells via STAT3^23^, though in a non-cancer derived HTR8 trophoblast cell line, LIF promoted proliferation via ERK1/2, but not STAT3^24^.

Given the pattern of localization in the human placenta and *in vitro* function in trophoblast cells, we hypothesised that LIF is also present in the mouse placenta and required for normal placental development *in vivo*. To address this hypothesis, we determined the effect of transient LIF blockade during placental formation in mice and the effects on pregnancy outcome.

## Results

### LIF and LIFRα Expression and Localization in the Mouse Placenta

**Figure 1:**
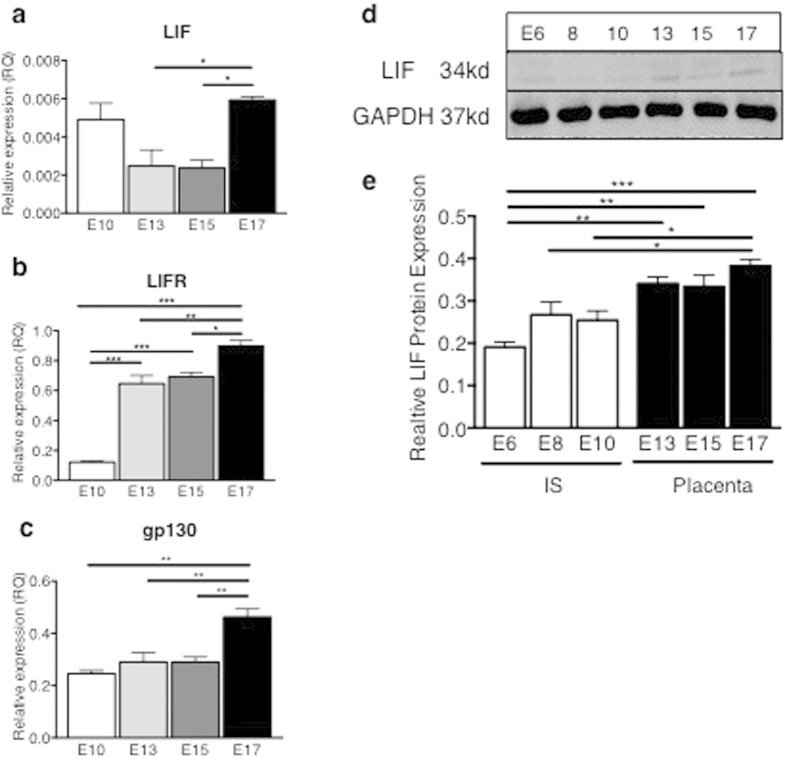
LIF and LIFRα expression in wild-type mouse implantation sites and placenta throughout gestation. Wild type (WT) mouse implantation sites were collected from n = 3 mice/timepoint. (**a**) LIF, (**b**) LIFRα and (**c**) gp130 mRNA expression were analysed in E10, 13, 15 and 17 placenta by quantitative real-time PCR, normalized to β2-microglobulin. Data are mean ± SEM, ANOVA, Tukey’s post-hoc test, *p < 0.05, **p < 0.01, ***p < 0.001. (**d**) LIF protein expression was analysed in E6, 8, 10 implantation sites and E13, 15 and 17 placenta by Western blot, normalized to GAPDH. (**e**) Data are mean ± SEM, ANOVA, Tukey’s post-hoc test, *p < 0.05, **p < 0.01, ***p < 0.001.

**Figure 2:**
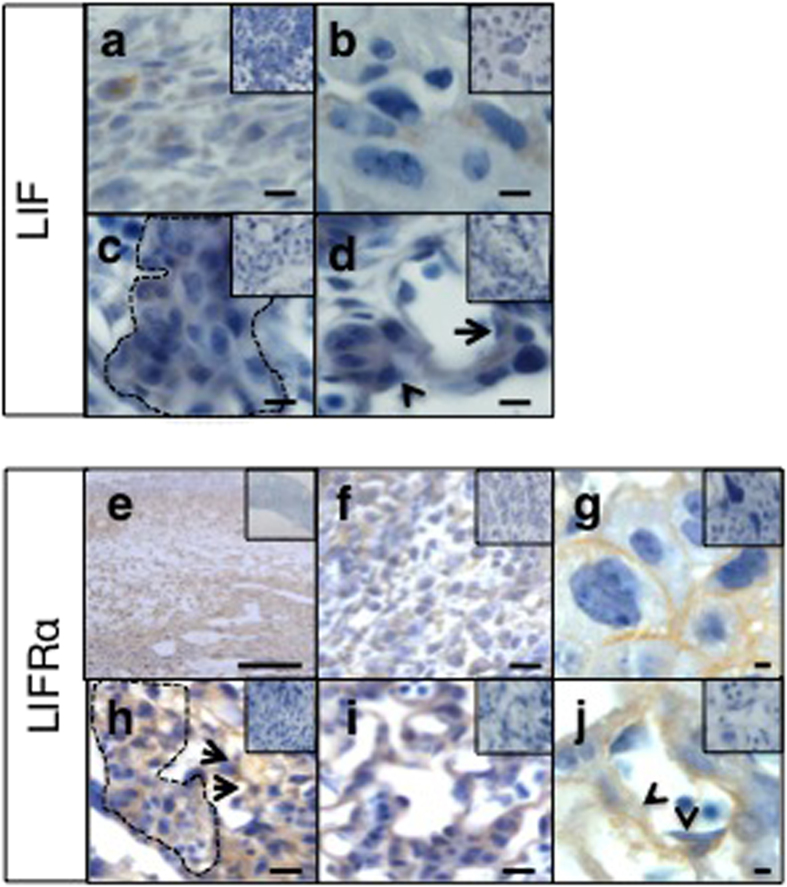
LIF and LIFRα localization in wild-type mouse implantation sites. Wild type (WT) mouse implantation sites were collected from n = 3 mice/timepoint and immunostained for LIF and LIFRα. Representative photomicrographs of mid-gestation (E13) implantation site sections are shown here. (**a**) LIF localized to decidual cells, (**b**) trophoblast giant cells, (**c**) spongiotrophoblast islands (line) and (**d**) some mononuclear trophoblasts associated with maternal vascular spaces (arrow) as well as endothelial cells lining fetal cappillaries (arrow head). (**e**) LIFRα was produced abundantly throughout the implantation sites. (**f**) LIFRα localized to decidual cells. In the junctional zone LIFRα was produced by (**g**) trophoblast giant cells, (**h**) spongiotrophoblast islands (line) and opposing glycogen cells (arrows). In the placental labyrinth, LIFRα localized to (**i**) syncytiotrophoblasts and mononuclear trophoblasts associated with maternal vascular spaces and also (**j**) endothelial cells lining fetal cappillaries (arrow heads). Bars represent 20 μm (**a–d, f–j**) and 200 μm (**e**). Insets are negative controls.

To help determine the functional role of LIF during placental development, we investigated the expression and localization LIF and LIFRα in whole implantation sites (containing decidua and early placenta) during early-gestation (E6–10) and the mouse placenta alone from mid-gestation onwards (E13–17). LIF mRNA levels were maximal during late-gestation at E17 in the mouse placenta, with moderate expression during mid-gestation (Fig. 1a). Similarly, LIFRα and gp130 mRNA expression progressively increased across gestation (Fig. 1b,c). LIF protein was quantified by Western blotting and levels were increased during mid-late gestation (E13–17) during placental development, compared to early gestation (E6–10) (Fig. 1d,e), reflecting transcript levels. In mouse implantation sites, LIF localized to decidual cells, trophoblast giant cells, spongiotrophoblast islands and mononuclear trophoblasts associated with maternal vascular spaces, as well as endothelial cells lining fetal capillaries (Fig. 2a–d). LIFRα was produced abundantly throughout the implantation sites and localized to decidual cells. In the junctional zone LIFRα was produced by trophoblast giant cells, spongiotrophoblast islands and opposing glycogen cells. In the placental labyrinth, LIFRα localized to syncytiotrophoblasts and mononuclear trophoblasts associated with maternal vascular spaces and also endothelial cells lining fetal capillaries (Fig. 2e–j).

### Blocking LIF Action During Placentation Altered Placental Morphology & Reduced Pregnancy Viability

**Figure 3:**
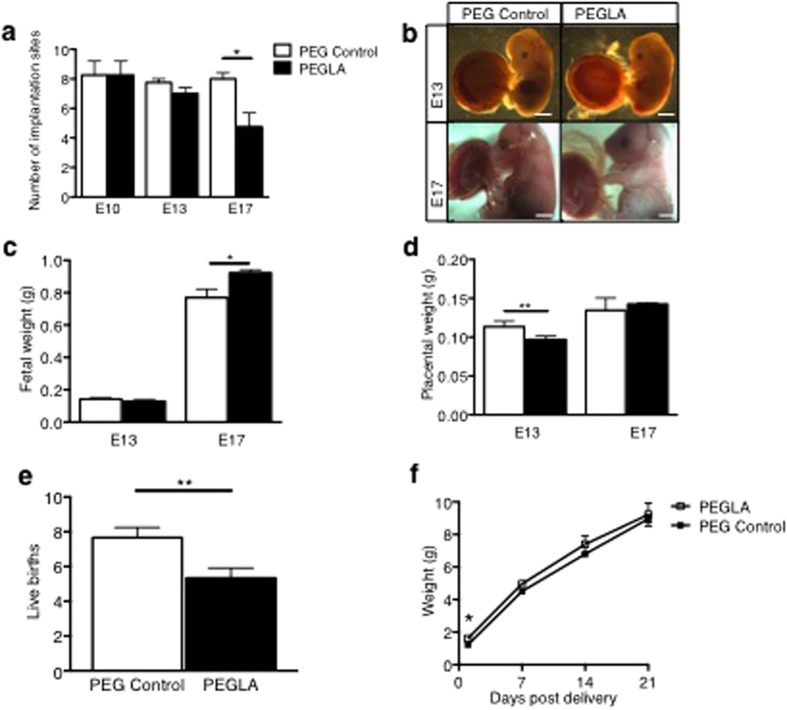
LIF inhibition during placental development reduces pregnancy viability in mice. (**a**) C57BL/6 mice were treated with PEG vehicle control or PEGLA (20 mg/kg/day) from E8–10, E10–13 or E10–17 (n = 4/group) and whole implantation sites collected and counted at E10, 13 or 17 respectively. **(b–d)** Fetal and placental weights were obtained (grams). (**e**) A second group of mice treated from E10–17 were left until term and the number of live pups recorded. (**f**) Pup weights were obtained at D1, 7, 14, 21 post delivery. All data are mean ± SEM, students t-test, *p < 0.05, **p < 0.01.

**Figure 4:**
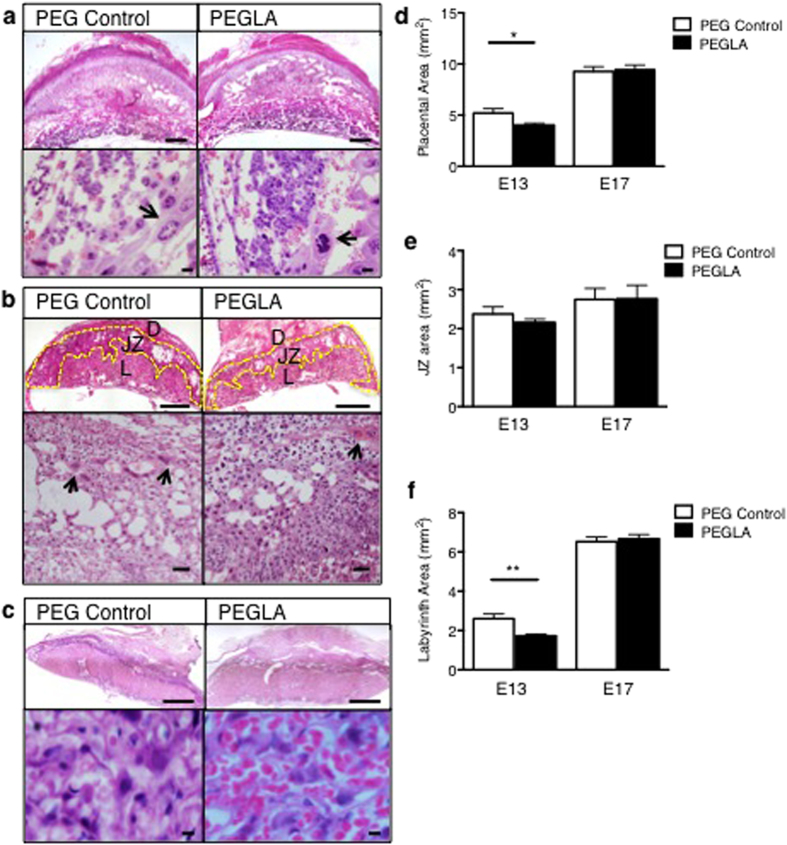
LIF inhibition during placental development results in abnormal placental morphology in mice. E10, 13 or 17 implantation sites treated with PEG control or PEGLA from E8–10, E10–13 or E10–17 were stained with haematoxylin-eosin (H&E). **(a)** At E10 (**b**) E13 and (**c**) E17, low and high power images of the implantation sites are shown. Arrows denote trophoblast giant cells (top panel, bars represent 500 μm, lower panel, bars represent 50 μm). (**b**) At E13, the deciua (D), spongiotrophoblast (yellow outline, S) and labyrinth (L) are denoted (top panel, bars represent 500 μm, lower panel, bars represent 500 μm). (**d–f**) At E13 and E17, the total placental, spongiotrophoblast (junctional zone, JZ) and labyrinth area (mm^2^) were calculated using CellSense software. At least 3 mid-sagital sections of implantation sites per mouse (n = 4/group) were analysed. Data are mean ± SEM, students t-test, *p < 0.05.

In order to determine the role of LIF during placentation, which occurs maximally during mid-gestation from E8-E13^25^, mice were treated with PEG vehicle control or PEGLA from either E6–8, E8–10, E10–13 or E10–17 to block LIF action. Mice were sacrificed on the final day of PEGLA treatment or allowed to continue pregnancy to term. PEGLA treatment did not affect maternal weight (Supp Fig. 1). At E10 and E13, there were no differences in the number of viable or resorbed implantations sites between groups, however by E17, there was significant pregnancy loss in PEGLA treated mice (PEG 8.00 ± 0.41 vs. PEGLA 4.75 ± 0.95) (Fig. 3a), which was maintained at birth (Fig. 3e). Fetal weight was significantly increased in PEGLA mice at E17 (PEG 0.769g ± 0.052 vs. PEGLA 0.922g ± 0.019) (Fig. 3c) and also at birth, but normalized to control weight by weaning (Fig. 3f). Placental weight was significantly reduced in PEGLA mice at E13 (PEG 0.113g ± 0.003 vs. PEGLA 0.096g ± 0.002), but similar to control by E17 (Fig. 3d). While the decidua was unaffected by LIF blockade (Supp Fig. 3), placental morphology was altered at all time points (Fig. 4). In mice treated with PEGLA from E6–8, E8–10 or 10–13, trophoblast giant cells appeared abnormal and reduced in number at E10 and E13 respectively, compared to control (Fig. 4a,b; Supp Figure 2). At E13, total placental area was significantly reduced in PEGLA treated mice (4.02 mm^2^ ± 0.07) compared to control (5.45 mm^2^ ± 0.40). Specifically, there was a significant reduction in the labyrinth area (PEGLA 1.73 mm^2^ ± 0.16 vs. PEG 2.62 mm^2^ ± 0.27), but no differences in the junctional zone (spongiotrophoblast) (Fig. 4d–f). By E17, although placental area was unchanged between treatment groups (Fig. 4d–f), haematoxylin and eosin staining revealed morphological differences in the placental labyrinth following LIF-inhibition (Fig. 4c). There was an apparent increased influx of red blood cells in the PEGLA-treated placental labyrinth compared to control, although this was not quantified (Fig. 4c).

### LIF Inhibition Reduced Activated STAT3, but not ERK

**Figure 5:**
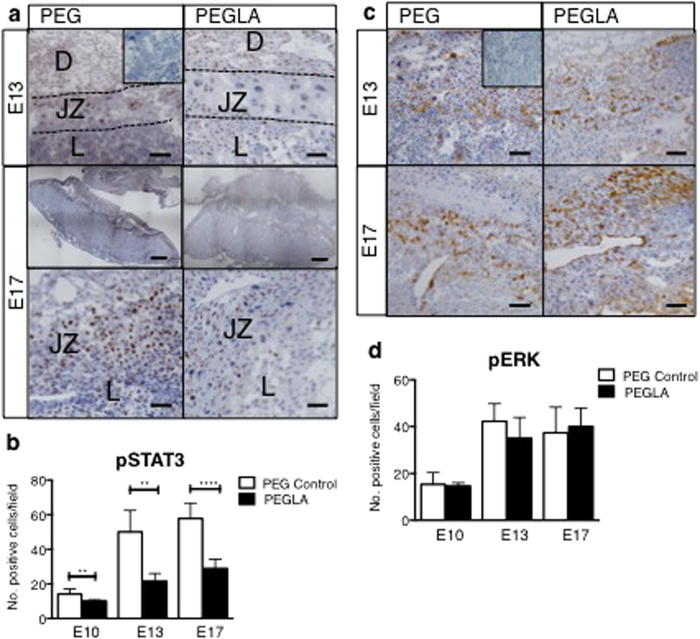
LIF inhibition during placental development reduces activated STAT3, but not ERK in the mouse placenta. Representative photomicrographs E10, 13 or 17 implantation sites treated with PEG control or PEGLA from E8–10, E10–13 or E10–17 stained for (**a**) phosphorylated (p)STAT3 or, **(c)** pERK1/2. Bar represents 100 μm. Insets are negative controls. (Decidua, D; Junctional Zone, JZ; Labyrinth L). (**b,d**) The number of positive cells (brown) were counted in at least 4 fields of 3 mid-sagital implantation site sections per mouse (n = 4/group) and averaged. Data are mean ± SEM, students t-test, **p < 0.01, ***p < 0.001.

In PEG-treated mouse implantation sites, STAT3 is activated in decidual cells, trophoblast giant cells, spongiotrophoblast cells and some trophoblast subtypes in the labyrinth (Fig. 5a). Blocking LIF action in the mouse placenta significantly reduced the number of pSTAT3 positive cells in all of these cell types at E10, 13 and 17 (Fig. 5a,b). Conversely, PEGLA had no effect on the number of pERK1/2 positive cells in the mouse implantation sites (Fig. 5c,d).

### LIF Blockade Alters Placental Trophoblasts

**Figure 6:**
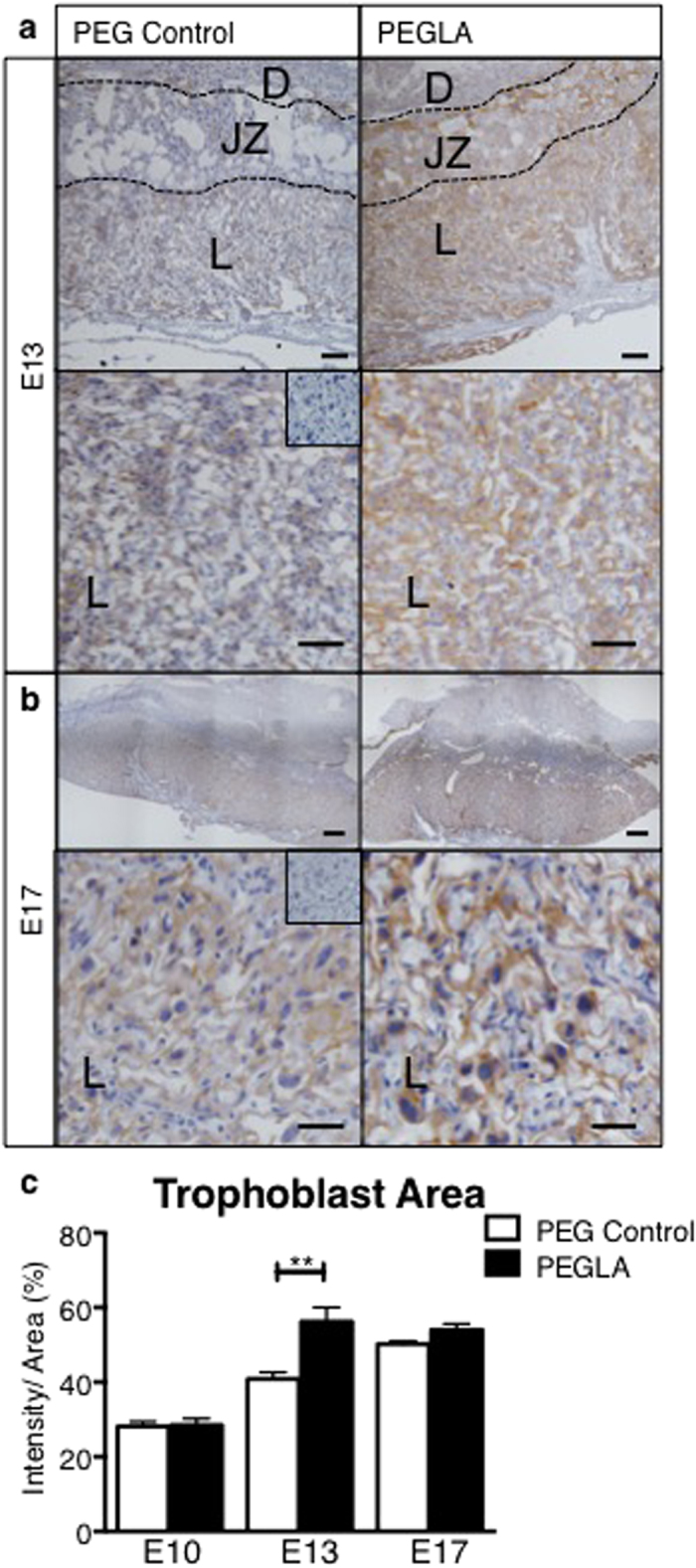
LIF blockade during placentation increases trophoblast area at mid-, but not late-gestation. Representative photomicrographs of **(a)** E13 or **(b)** E17 implantation sites treated with PEG control or PEGLA from E10–13 or E10–17 stained for cytokeratin. **(c)** Cytokeratin staining intensity was analysed using CellSense software and quantified as staining intensity (pixels) per area (%) in at least 4 fields of 3 mid-sagital implantation site sections per mouse (n = 4/group) and averaged. Bars represent 50 μm, inset is negative control. Data are mean ± SEM, students t-test, **p < 0.01.

Given that LIF and LIFRα localized to the mouse placental trophoblasts and vasculature, the effects of PEGLA on these cell types was investigated. The area of cytokeratin-positive staining in the placenta, including the junctional zone and labyrinth, was quantified. There were no differences in placental trophoblast area at E10. However we found a significant increase in trophoblast area at E13 in PEGLA treated placenta (57.88% ± 4.92) compared to control (39.45% ± 1.87) (Fig. 6a). While this quantification included both the junctional zone and labyrinth, morphologically, differences were most evident in the labyrinth. At E17, there were no differences in trophoblast area between treatment groups (PEG 50.21% ± 0.81; PEGLA 54.01% ± 1.61) (Fig. 6b).

### LIF Blockade Impairs Placental Vascularization *In Vivo* and Alters Mouse Placental Angiogenic/Vascular and Oxidative Stress Gene Transcription

**Figure 7:**
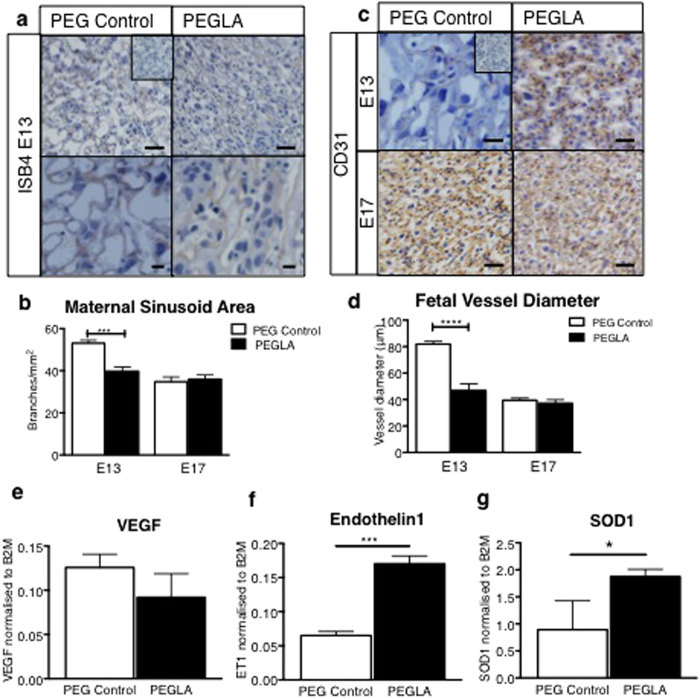
LIF inhibition during placental development impairs placental vasculature development and alters mouse placental angiogenic/vascular and oxidative stress gene transcription. Representative photomicrographs E13 or 17 implantation sites treated with PEG control or PEGLA from E10–13 or E10–17 stained for (**a**) Isolectin-B4 to highlight extracellular matrix surrounding maternal sinusoid branches, which were counted (**b**), or (**c**) CD31 immunostaining to highlight fetal vessel endothelial cells. Bar represents 100 μm. Insets are negative controls. (**d**) The fetal vessel diameter was measured in at least 4 fields of 3 mid-sagital implantation site sections per mouse (n = 4/group) and averaged. Data are mean ± SEM, students t-test, ***p < 0.001. (**e–f**) Candidate genes from a PCR array screen (Supp Table 1) on E13 PEG or PEGLA mouse placenta were validated by semi-quantitative PCR and normalized to β2-microglobulin (n = 4/group). Data are mean ± SEM, students t-test, *p < 0.05, ***p < 0.001.

Dramatic differences in the mouse placental labyrinth vasculature were also found following PEGLA treatment. Isolectin-B4 staining highlighted a significant reduction in the maternal blood sinusoid branching area in the labyrinth of PEGLA treated mice (58.83 branches ± 2.43) compared to control (38.33 branches ± 1.72) at E13 (Fig. 7a,b). Similarly, at E13, CD31 immunostaining revealed a significant reduction in labyrinth fetal vessel diameter of PEGLA treated mice (81.58 μm ± 2.83) compared to control (50.03 μm  ± 6.02). However, by E17 there were no differences between PEGLA treated mice (34.75 branches ± 2.25) compared to control (36.00 branches ± 1.99) (Fig. 7b–d). To determine how blocking LIF contributes to these morphological changes, we investigated target genes in the mouse placenta using QIAGEN RT^2^ Profiler Mouse Preeclampsia PCR Arrays. PEGLA up-regulated 12 target genes by >2-fold and down-regulated 3 target genes by >2-fold (Supp Table 1). Most gene changes found are involved in the regulation angiogenesis and vascular function, including SOD-1, EDN-1, FLT-1, ANGPT2 and HSP90. Of these, we validated SOD-1 and EDN-1, which were increasing in PEGLA versus control group and also VEGF (which is antagonized by FLT-1), which was unchanged (Fig. 7e–g). Other notable gene changes observed included genes involved in the regulation of metabolism, cell growth and immune and ECM factors (Supp Table 1). The transcript levels of LIFR and gp130 and the IL6 family cytokines including LIF, IL11 and IL6 were unchanged between groups, suggesting that there is no compensation among these family members (Supp Fig. 3).

### LIF Inhibition Promotes Decidual Apoptosis During Mid-gestation Pregnancy in Mice, but Does not Impair Decidualization

**Figure 8:**
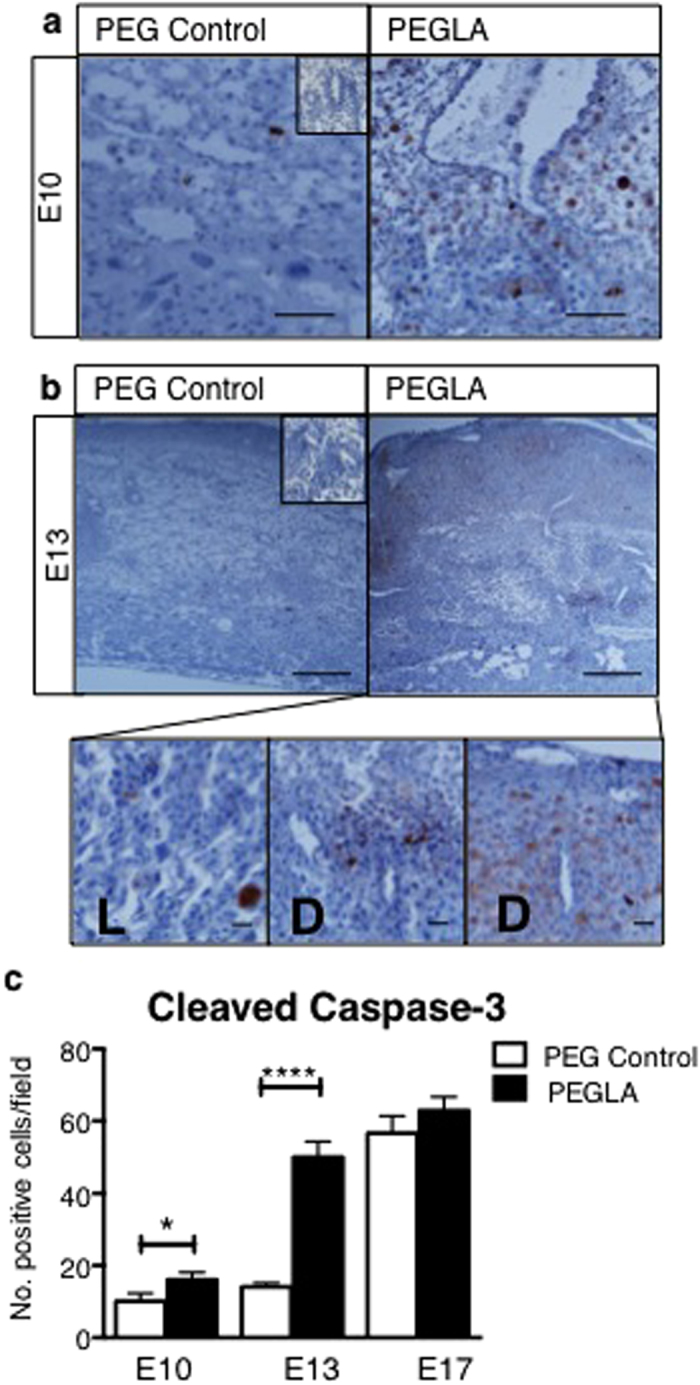
LIF inhibition during placental development promotes decidual apoptosis. Representative photomicrographs **(a)** E10 or **(b)** E13 implantation sites treated with PEG control or PEGLA from E8–10 or E10–13, immunostained for cleaved caspase-3. **(b)** At E13, high power images of apoptotic cells in PEGLA-treated placental labyrinth (L) and decidual-placental interface (D) are shown. Bars represent 100 μm. Insets are negative controls. The number of positive cells (brown) were counted in at least 4 fields of 3 mid-sagital implantation site sections per mouse (n = 4/group) and averaged. Data are mean ± SEM, students t-test, *p < 0.05, ****p < 0.0001.

To determine how LIF inhibition may contribute to pregnancy loss *in vivo,* we immunolocalized cleaved caspase-3-positive apoptotic cells in PEG vehicle control or PEGLA-treated implantation sites. At E10 the number of cleaved caspase-3 positive cells was significantly increased in PEGLA treated implantation sites (16.13 ± 1.99) compared to PEG control (10.13 ± 0.76) (Fig. 8a,c) and this was greatly enhanced by E13 (PEG 14.13 ± 1.04; PEGLA 50.00 ± 4.39) (Fig. 8b,c). The apoptotic cells were primarily localized to the placental-decidual interface at E10 and similarly at E13, with some apoptosis also in the PEGLA-treated placental labyrinth (Fig. 8a,b). However, by E17 there were large numbers of decidual apoptotic cells in both treatment groups (PEG 56.63 ± 4.82; PEGLA 62.88 ± 3.81) (Fig. 8c). Despite this, decidual area (quantified desmin immunostaining) was unchanged between treatment groups at all time points (Supp Fig. 4).

## Discussion

This study is the first to determine the role of LIF in mouse placental development *in vivo* and to localize and determine the expression levels of LIF and LIFRα in the mouse placenta throughout gestation. LIF and its signalling components LIFRα and gp130 were all expressed in the mouse placenta at the transcript level during placentation. LIF protein was also produced in increased levels during mid- compared to early-gestation. The localization pattern of LIF was restricted to a few trophoblast subsets and endothelial cells within the mid-gestation mouse placenta. By temporally blocking LIF action during the initiation of placentation in mice using our specific LIFRα antagonist, PEGLA, we demonstrated that LIF is required for normal placental development and morphology at mid-gestation and vital for pregnancy viability in mice.

Given that the LIFRα is expressed abundantly throughout the implantation site, this suggests that LIF could act in an autocrine and/or paracrine manner at this site. Similarly, in women the high levels of LIF expression in first trimester decidua and the localization of its receptor on trophoblast cells suggests paracrine actions during placentation^20^,^21^. Here we show that LIF inhibition reduced total placental area and also labyrinth area. The labyrinth is the crucial site of fetal-maternal exchange in the mouse placenta and analogous to the human chorionic villous^25^. We found dramatic alterations in placental trophoblast and vascular morphology at mid-gestation. The trophoblast area was significantly increased in the PEGLA group compared to the PEG control group. Conversely, fetal and maternal vessel size was significantly reduced in the PEGLA group, which could compromise fetal-maternal exchange^25^,^26^,^27^.

Consequently, we found reduced pregnancy viability in mice treated with PEGLA from E10–17. The number of implantation sites at late-gestation and also litter size at birth was significantly reduced, we observed 40% pregnancy loss in PEGLA-treated mice. This was potentially facilitated by an increase in decidual apoptosis during early- and mid-gestation in PEGLA-treated mice, which is know to contribute to pregnancy loss in mice^28^ and humans^29^,^30^. There were no differences in the number of apoptotic cells at E17 between treatment groups, likely due to the increase in apoptosis in control implantation sites that occurs naturally to facilitate parturition. Interestingly, the surviving fetuses were healthy at birth and until weaning and weighed more than control pups. We speculate that this could be attributed to compensatory increased blood and nutrient supply to the few surviving implantation sites, although uterine artery flow was not quantitated.

In PEGLA treated mouse implantation sites, obvious abnormalities were evident particularly in the trophoblast giant cells with PEGLA treatment from E6–8, E8–10 and also E10–13. Trophoblast giant cells are the first trophoblast cell subtype to terminally differentiate in mice and are vital for placentation^31^. Trophoblast giant cells secrete hormones and paracrine factors to target the maternal physiological systems for proper maternal adaptations to pregnancy, and target the fetal-maternal interface to ensure vasculature remodeling^31^. The morphological observations demonstrated in this study are consistent with previous findings that have shown administration of recombinant LIF protein promotes trophoblast giant cell differentiation *in vitro,* and *in vivo* and genetic reduction in LIF in SOCS3-null mutants, rescues trophoblast giant cells phenotype suggesting that this was due to un-regulated LIF signalling^32^,^33^. However, LIF production and localization was not investigated in those studies, so the mechanisms of action in these specific cells could not be determined^32^,^33^. Similar to our observations, in LIFR-mutants trophoblast giant cell numbers are reduced, and spongiotrophoblast and labyrinth layers are also disorganized^19^,^33^. From the findings in the present study, it is now clear that LIF and LIFRα both localize to trophoblast giant cells in the mouse placenta. Given that PEGLA reduced pSTAT3 in these cells, this supports and strengthens our data that LIF-STAT3 signalling is crucial for the development and differentiation of this cell type *in vivo*.

In the human endometrium and primary human trophoblasts, LIF acts via STAT3^22^,^34^. Here we found that PEGLA reduced activated STAT3, but not ERK1/2 positive cells, suggesting that LIF acts predominantly via STAT3 in the mouse placenta *in vivo,* similar to primary first trimester trophoblasts *in vitro*^22^. PEGLA treatment reduced STAT3 in decidual cells, trophoblast giant cells, spongiotrophoblast cells and some trophoblast subtypes in the labyrinth, suggesting that LIF signalling is important in these cell types in the mouse placenta. In accordance, we also observed morphological alterations in these same cell types following LIF inhibition.

We previously demonstrated that LIF promotes decidualisation in mice early post-implantation E4-5^35^. In the present study, we observed no decidual alterations following PEGLA. However, this is likely due to the fact that mice were administered with PEGLA from E8 of gestation onwards, after the time of critical establishment of the maternal decidua in mice^25^. Previous findings also demonstrate LIF-mediated regulation of ECM factors critical for endometrial remodeling during early pregnancy^22^,^35^. Here we found gene changes in some ECM factors important for trophoblast invasion and migration at the maternal-fetal interface including MMP9, which was the most highly up-regulated gene in PEGLA treated placenta versus control at mid-gestation. MMP9-null mice exhibit impaired trophoblast invasion^36^, suggesting that this protease promotes invasion. In contrast, previous findings from our research group have shown that exogenous LIF increases first trimester primary human trophoblast invasion and also that PEGLA can block this effect^37^. In support, in women, strong expression of LIF mRNA has been detected in decidual leukocytes, which are abundant at the implantation site, suggesting that LIF may mediate interactions between maternal decidual leukocytes and invading trophoblast cells^20^. Although trophoblast invasion specifically was not investigated here, this could be an avenue for future investigation to determine whether *in vivo* and *in vitro* findings are reconciled.

LIFR-knockout mice are perinatal lethal^19^, while LIF-deficient female mice are infertile due to implantation failure, though it is clear from embryo transfer studies from LIF null-mice into WT mothers, that only maternal LIF is required for normal implantation^15^,^17^. Here, we found no alterations in IL11 or IL6 transcript levels following LIF blockade, suggesting non-redundancy amongst these family members. However, LIF and oncostatin-M (OSM) share the LIFR signalling subunit. While there may be some redundancy between these family members^38^, the phenotype of OSM knockout mice does not exhibit pregnancy defects^39^. In this study, LIF blockade from E10 onwards did not result in perinatal lethality, suggesting the defect seen in LIFR-null mice may occur earlier and/or more likely be attributed to loss of fetal, not placental LIF signalling. Indeed in LIFR-mutant fetuses, skeletal and neuronal defects were evident during embryogenesis^19^. PEGLA is a large molecule of >100 kDa^13^ and our data suggests that very little crosses the placenta (unpublished data), so in this study we were able to exclusively target LIF signaling in the placenta with minimal effects on fetal development during mid-gestation.

The results from this study support an important role for LIF in the maintenance of healthy pregnancy. Recurrent pregnancy loss affects about 1–5% of women who conceive^40^. In up to 50% of patients who experience pregnancy loss, the underlying causes remain undetermined^41^ and is therefore an important area of research. Abnormalities of placental vasculature are thought to result in several gestational complications, including pregnancy loss, intrauterine fetal death, intrauterine growth restriction and preeclampsia^42^,^43^. Microarray data have shown a decreased expression pattern of angiogenesis-related genes in the chorionic villi of recurrent pregnancy loss patients^44^.

A striking defect observed in PEGLA treated mouse placenta was the impaired labyrinth vascular development at mid-gestation. There is conflicting data on the role of LIF in angiogenesis. The retinas of LIF-deficient mice display increased microvessel density due to increased VEGF expression in the vascularized area^45^. However, exogenous LIF inhibits VEGF-mediated angiogenesis *in vitro* in bovine aortic endothelial and bovine microvascular endothelial cells^46^. In the present study, VEGF, a driving angiogenic factor, showed a trend to reduced transcript levels. Although its levels did not change significantly following PEGLA in the mouse placenta, mRNA expression for the VEGF antagonist, FLT-1 (VEGFR1) was significantly up-regulated in PEGLA-treated placenta versus PEG control. In order to definitively determine the effect of LIF on placental vascularization, it would be interesting to use a 3-dimensional vascular cast approach^47^, however using cross-sectional analysis, the fetal and maternal vascular spaces showed significantly reduced area in PEGLA-treated mice compared to PEG control. In support, endothelin-1 mRNA was significantly up-regulated following LIF inhibition. Endothelin-1 localizes to fetal vessels in the mouse placental labyrinth and plays a functional role in promoting vasoconstriction^48^, which may reduce maternal-fetal exchange.

Conventionally, impaired maternal-fetal exchange can result in oxidative stress, pregnancy loss and intrauterine growth restriction in surviving fetuses^49^, resulting from an imbalance between reactive oxygen species (ROS) and the cell’s antioxidant capacity^50^. The enzymatic defense of cells against ROS involves the cooperative action of several enzymes, including SOD^51^, an enzyme that catalyses superoxide conversion into H_2_O_2_. LIF is protective against oxidative stress in kidney podocytes^52^. In support, in the mouse placenta, we found that SOD-1, and also heat shock protein (HSP90) transcript levels, which are associated with oxidative stress, were up-regulated in response to LIF blockade, suggesting a state of placental oxidative stress in these mothers. Conversely, the increased fetal and pup weight of PEGLA litters could be attributed to a compensatory effect, since these gene changes were observed at mid-gestation. Due to the increased number of resorbed embryos, the maternal blood supply is most likely directed to a smaller number of implantation sites and in rodents, utero-placental blood flow is strongly correlated with fetal weight^53^. Indeed, we observed increased RBC accumulation in PEGLA placentas at late gestation, however utero-placental blood flow was not quantified. The pregnancy loss observed in PEGLA treated mice occurred beyond E13, since there were no differences in the number of viable embryos at this time. In an LPS-induced pregnancy loss model, administration of recombinant LIF, combined with progesterone was able to maintain pregnancy in mice^54^. The authors of this study proposed an anti-inflammatory effect of LIF. Here we found that transcript levels of inflammatory mediators that promote immune cell recruitment, including SPP-1 and CCL12 were increased in response to reduced LIF signalling, although hormonal factors were not investigated here.

## Conclusion

In this study we have demonstrated that targeted inhibition of placental LIF signalling lead to abnormal placental morphology and increased pregnancy loss, likely due to trophoblast and/or vasculature defects. We have uncovered the possible mechanisms by which LIF regulates placentation *in vivo* in mice. These data strengthen the rationale that LIF is a critical player in the establishment and maintenance of placentation and a healthy pregnancy.

## Materials and Methods

### Animals and Wild-type Implantation Site Tissue Collection

Female (virgin 8–12 weeks old) and male C57BL/ 6J mice (Monash Animal Services, Clayton, Australia) were housed under conventional conditions, with food and water available *ad libitum* and held in a 12 hr light and dark cycle. All procedures were approved by the Monash Medical Centre Animal Ethics Committee (#MMCB/2012/17) and followed the NHMRC Australian Code of Practice for the Care and Use of Animals for Scientific Purposes. Implantation sites were collected from wild-type (WT) mated female mice on embryonic days (E) 6–17 of gestation (E0 = day of plug detection) (n=3 mice/time point).

### PEGLA Treatments and Tissue Collection

We produced polyethylene glycol (PEG) conjugated LIF-antagonist (PEGLA)^13^ as previously described. The LIF antagonist binds to the LIF receptor but does not bind to the LIF receptor complex signalling component, gp130, preventing the initiation of downstream signalling. LA was covalently bound to PEG (PEGLA) to increase the period of sera retention^49^. To inhibit LIF action in the placenta, mated female mice were injected IP with 600 μg/dose PEGLA (20 mg/kg/dose) or equal molarity PEG as a control (as optimized previously based on serum half life studies and *in vivo* dose response studies^18^,^35^,^55^, twice daily at E6–8, E8–10, E10–13 or E10–17) (n = 4 mice/group). Mice were weighed and killed at E10, E13 or E17 of pregnancy respectively, or allowed to deliver at term. Uterine horns were removed and the number of implantation sites counted. Implantation sites containing the placenta (separated from the maternal decidua) and fetus were dissected out and weighed, then either fixed in 10% neutral buffered formalin (18 hrs) or snap frozen.

### RNA Extraction and Polymerase chain reaction

Total RNA was isolated from snap frozen placental tissue using the RNeasy Minikit (QIAGEN) according to the manufacturer’s instructions. Genomic DNA was digested using the DNAfree kit (Ambion) according to the manufacturer’s instructions. RNA samples were analysed by spectrophotometry (Nanodrop) at an absorbance ratio of A260/280 nm to determine RNA concentration, yield and purity. cDNA was synthesized from total RNA (500 ng) using Superscript III reverse transcriptase (Invitrogen) and analyzed by spectrophotometry at an absorbance ratio of A260/280 nm to determine concentration and purity. PCR reactions were performed using PCR express machine (Thermo Fisher Scientific) and GoTaq master mix (Promega) according to the manufacturer’s instructions. cDNA was analysed for LIF, LIFR, gp130, VEGF, SOD1, Endothelin-1, IL11, IL6 and β2-microglobulin using reaction conditions of an initial denaturation at 95 °C for 3 mins, followed by 30 cycles of: denaturation, 95 °C for 30 secs; annealing, 60 °C for 30 secs; extension, 72 °C for 1 min; with a final extension at 72 °C for 10 min. Primers used were validated and are detailed in Supplementary Table 2 with calculated efficiency (%). The PCR products were run on a 1.5% agarose gel with 1000 bp DNA ladder (Invitrogen) to semi-quantify gene expression.

### PCR Arrays

Screening for the expression of 84 genes associated with the mouse placenta in PEGLA or PEG control mouse placental samples at E13 (n = 3/group) was conducted using the QIAGEN RT^2^ Profiler Mouse Preeclampsia PCR Arrays, according to the manufacturer’s instructions and our previous studies^22^. In brief, cDNA was prepared from 500 ng total RNA by using a RT2 PCR array first strand kit. PCR amplification was conducted with an initial 10-min step at 95 °C followed by 40 cycles of 95 °C for 15 s and 60 °C for 1 min. The fluorescent SYBR Green signal was detected immediately after the extension step of each cycle, and the cycle at which the product was first detectable was recorded as the cycle threshold. Data were imported into an Excel database and analyzed using the comparative cycle threshold method with normalization of the raw data to β2-microglobulin.

### SDS-PAGE and Western blotting

Whole tissues were lysed in ice-cold lysis buffer (50 mM Tris-HCl (pH 7.5), 150 mM NaCl, 2 mM EDTA, 2 mM EGTA, 25 mM NaF, 25 mM β-glycerolphosphate and protease inhibitor cocktail (Calbiochem) and the protein was quantified by Bradford assay. Equal amounts of protein per sample were resolved on 8–10% sodium dodecyl sulfate (SDS)–polyacrylamide gel electrophoresis (PAGE) gels, transferred to polyvinyl difluoride (PVDF) membranes (GE Healthcare Bio-Sciences), blocked with 5% non-fat dry milk in Tris-buffered saline (TBS) with 0.1% Tween-20 (Bio-Rad) and probed with polyclonal antibody against LIF (1:500; R&D Systems, #AF-250NA) overnight at 4 °C, followed by three wash steps. Membranes were incubated 1 h at room temperature with secondary anti-rabbit IgG-horse-radish peroxidase (HRP) linked, (1:5000; Dako) and signals were developed with enhanced chemiluminescence Western blotting detection system reagent (Pierce). Membranes were stripped and incubated with anti-GAPDH as a protein loading control.

### Histological and Immunohistochemical Staining

Paraffin embedded mouse implantation sites (n = 3/wild-type gestation day, or n = 4/treatment group) were sectioned (5 μm), dewaxed in histosol (Sigma-Aldrich) and rehydrated in a graded series of ethanol. Haematoxylin and eosin staining was preformed as previously described^13^. For immunohistochemistry, sections were microwaved at high power (700 W) in 0.01 M citric acid buffer (pH 6.0) for 5 min. Endogenous peroxidase activity was quenched with 6% H_2_O_2_ in 100% methanol (1:1 v/v) for 10 min. Tissues were incubated with non-immune blocking solution (10% normal horse serum, 2% normal mouse serum) diluted in 1 ×Tris-buffered saline (TBS) for 30 min. Primary antibody for LIFRα (1:100; R&D Systems #AF-249NA), LIF (1:100; R&D Systems #AF-250NA), phospho-STAT3 (Tyr705) (1:100; Cell Signaling Technologies #9145), phospho-p44/42 MAPK (Erk1/2, Thr202/Tyr204) (1:100; Cell Signaling Technologies #4370), pan-cytokeratin (1:200; Santa Cruz #H-240), CD31 (1:100; Abcam #ab28364), desmin (1:100; Dako, #D-33), isolectin-B4 (ISB4) (5 μg/section; Sigma #L5391) or non-immune goat IgG (isotype negative control) in blocking solution were applied for 18 h and incubated at 4 °C. After stringent washing with 0.6% Tween 20 in TBS, antibody localization was detected by sequential application of biotinylated horse anti-goat IgG (1:200; Vector Laboratories Inc., Burlingame, CA) in blocking solution for 30 min and an avidin-biotin complex conjugated to HRP (Vectastain ABC Elite kite; Vector Laboratories, Burlingame, CA, USA). Protein was visualized as a brown precipitate using diaminobenzidine tetrahydrochloride substrate (Dako). Sections were counterstained with Harris hematoxylin (Sigma), dehydrated and mounted with DPX.

### Placental Morphometry

The mouse implantation site comprises three distinct morphological layers. The decidua is of maternal origin, therefore not included in the placental area measurement. The underlying two layers; the structural junctional zone (JZ) and opposing vascular labyrinth make up the placenta. The cross-sectional area of these two layers combined was measured to give the total ‘placental area’. Digital photographs at 1X were taken and CellSense software was used to measure total placental area, as well as labyrinth and junctional zone areas seperately. Three independent measurements were taken for each layer and section. In order to assay vessel density, hemisected placentas were stained with ISB4 and CD31 as above. Six to twelve photographs at 20X magnification were taken from two different sections (middle region) representing more than 90% of the labyrinth. The vessels were counted/measured using CellSense software. A blinded observer counted/measured the vessels and counted Cleaved Caspase-3 positive apoptotic cells. To quantify ‘trophoblast area’, cytokeratin positive brown staining intensity was measured by automated CellSense software to quantify pixel density. The data was expressed as intensity per total ‘placental area’ (described above) to give an overall percentage, which represents the area of the placenta occupied by trophoblast cells.

### Statistical analysis

GraphPad Prism 6.0 (GraphPad Software) was used for all statistical analyses. Data were analysed by students t-test when comparing two groups, or ANOVA followed by Tukey’s post-hoc test when comparing more than two groups. Data were expressed as the mean ± SEM. Differences were considered significant at p < 0.05.

## Additional Information

**How to cite this article**: Winship, A. *et al.* Blocking Endogenous Leukemia Inhibitory Factor During Placental Development in Mice Leads to Abnormal Placentation and Pregnancy Loss. *Sci. Rep.* **5**, 13237; doi: 10.1038/srep13237 (2015).

## Supplementary Material

Supplementary Information
