# Supplementary material for: Blocking Endogenous Leukemia Inhibitory Factor During Placental Development in Mice Leads to Abnormal Placentation and Pregnancy Loss
Source: Sci Rep. 2015 Aug 14;5:13237. doi: 10.1038/srep13237 (PMC4536525; doi:10.1038/srep13237)
Supplement: Supplementary Information [file srep13237-s1.pdf]

**Supplementary Information**

# Blocking Endogenous Leukemia Inhibitory Factor During Placental Development in Mice Leads to Abnormal Placentation and Pregnancy Loss

Amy Winship[1,2], Jeanne Correia[1], Tara Krishnan[1,3], Ellen Menkhorst[1], Carly Cuman[1], Jian-Guo Zhang[4,5], Nicos A. Nicola[4,5], Evdokia Dimitriadis[1,2*]

[1] MIMR-PHI Institute of Medical Research, 27-31 Wright St, Clayton, VIC, 3168, Australia.

[2] Department of Anatomy and Developmental Biology, Wellington Road, Monash University, Clayton, Victoria, 3800, Australia.

[3] Faculty of Medicine, Nursing & Health Sciences, Wellington Road, Monash University, Clayton, Victoria, 3800, Australia.

[4] The Walter and Eliza Hall Institute of Medical Research, 1G Royal Parade, Parkville, Victoria 3052, Australia.

[5] Department of Medical Biology, The University of Melbourne, Parkville, Victoria 3010, Australia.

# Supplementary Figure 1

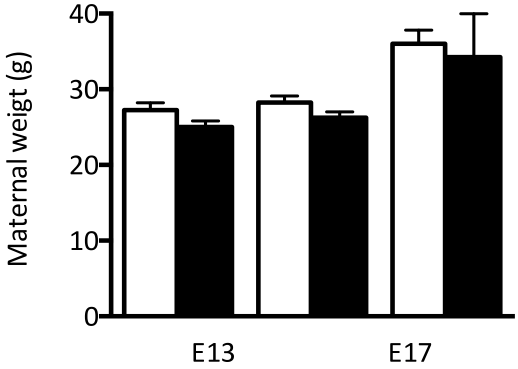


**Supplementary Figure 1. LIF inhibition during placental development did not change maternal weight.** Data are mean ± SEM.

# Supplementary Figure 2

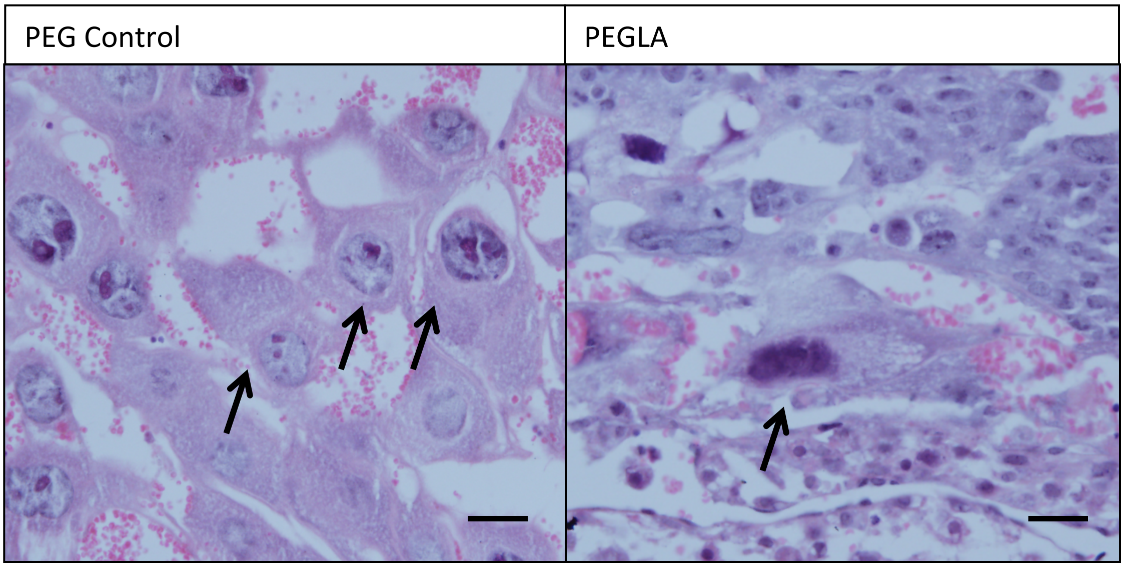


**Supplementary Figure 2. LIF inhibition from E6-8 altered trophoblast giant cell morphology in the mouse placenta at E10.** E10 implantation sites treated with PEG control or PEGLA from E6-8 were stained with haematoxylin-eosin (H&E). Abnormal trophoblast giant cell morphology is highlighted (Arrows). Bars represent 50μm.

# Supplementary Figure 3

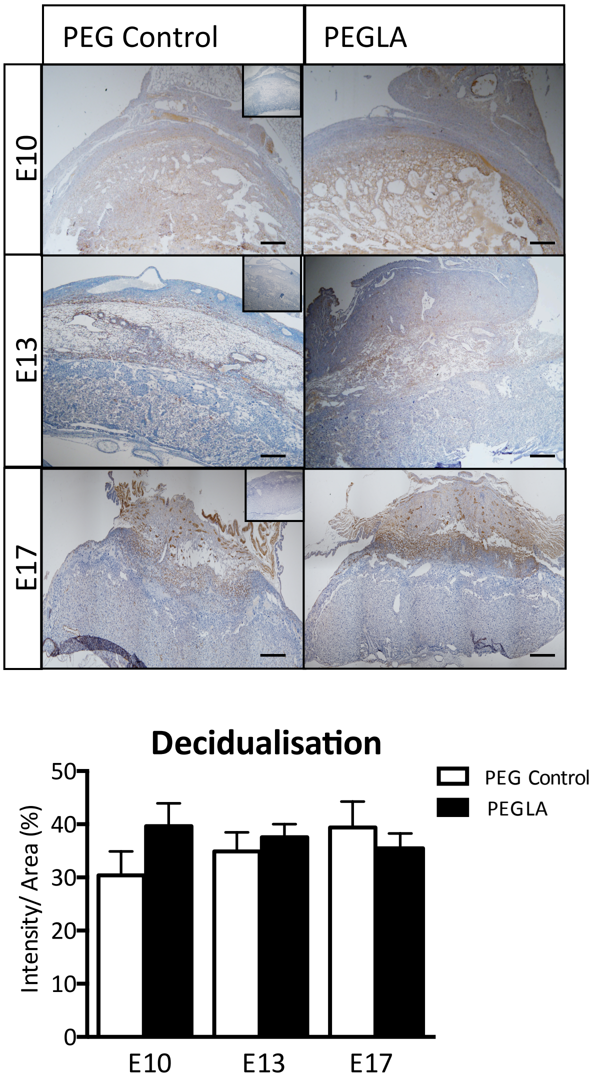


**Supplementary Figure 3. LIF inhibition during placental development did not affect decidualisation. (a)** Desmin immunostaining was performed to highlight the decidual area in E10, 13 or 17 implantation sites treated with PEG control or PEGLA from E8-10, E10-13 or E10-17. **(b)** Staining intensity was quantified as pixel intensity/area (%). Data are mean ± SEM.

# Supplementary Figure 4

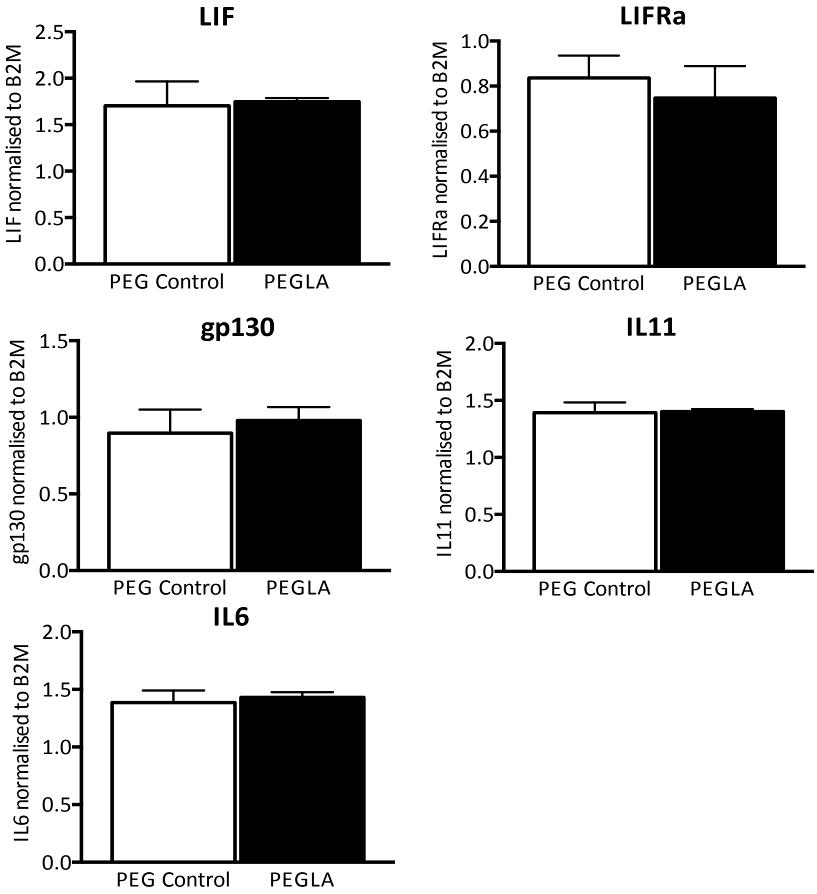


**Supplementary Figure 4. IL6 family gene expression levels were unchanged in PEG or PEGLA treated mouse placenta from E10-13, at E13.** LIF, LIFR, gp130, IL11 and IL6 mRNA expression were determined by semi-quantitative PCR and normalised to β2-microglobulin (n=4/group). Data are mean ± SEM.

**Supplementary Table 1. Effects of PEGLA and PEG control treatment from E10-E13 on mouse placental gene expression at E13.** Gene expression represented as genes up-regulated (+), or downregulated (-) by PEGLA versus control from highest to lowest abundance fold change from β2-microglobulin (n=4/group).

| Gene | Gene name | Fold change | P value | Function |
|---|---|---|---|---|
| ***Angiogenic & Oxidative Stress Factors*** | | | | |
| SOD1 | Superoxide dismutase-1 | +5.52 | 0.0189 | Reactive oxygen species scavenger, protects against oxidative stress |
| EDN-1 | Endothelin-1 | +4.25 | 0.0031 | Regulates vasoconstriction |
| FLT-1 | Fms-related tyrosine kinase 1 (VEGFR1) | +3.69 | 0.0215 | VEGF antagonist |
| ANGPT2 | Angiopoitin-2 | +3.55 | 0.0410 | Anti-apoptotic factor for stressed ECs in the placenta |
| HSP90 | Heat shock protein 90 | +2.16 | 0.0426 | Up regulated in response to EC stress in the placenta |
| CXCR4 | Chemokine receptor 4 | -3.23 | 0.0277 | Regulates VEGF synthesis |
| AGTR1A | Angiotensin-2 type 1a receptor | -2.00 | 0.0298 | Regulates blood pressure, vasodilation and fetal growth |
| ***Metabolic & Growth Factors*** | | | | |
| FABP4 | Fatty acid binding protein- 4 | +4.16 | 0.0195 | Regulates trophoblast triglyceride stores |
| IGFBP3 | Insulin growth factor binding protein 3 | +3.45 | 0.0218 | Trophoblast-derived protease |
| INHA | Inhibin-α | +2.01 | 0.0443 | Hormonal regulation |
| ***Immune Factors*** | | | | |
| SPP-1 | Osteopontin-1 | +7.07 | 0.0311 | Promotes macrophage recruitment |
| CCL12 | Chemokine ligand 12 | +3.53 | 0.0017 | Promotes neutrophil/monocyte recruitment |
| ***Extracellular Matrix Factors*** | | | | |
| MMP9 | Matrix metalloproteinase 9 | +7.95 | 0.0269 | Regulates trophoblast invasion |
| SERPINA3N | Serine peptidase inhibitor A3N | +6.22 | 0.0315 | Regulates trophoblast adhesion |
| VCAN | Versican | -2.00 | 0.0418 | Regulates trophoblast migration and promotes fibrosis |

**Supplementary Table 2. Primers used for gene transcription analysis.**

| Primer | Sequence: forward (F) and reverse (R) | Efficiency (%) |
|---|---|---|
| **LIF** | F 5'-TGAACCAGATCAGGAGCCT-3' | 89 |
| | R 5'-CCACATAGCTTGTCCAGGTTGTT-3' | |
| **LIFR** | F 5'-GTGGCAGTGGCTGTCATTGTTGGAGTGGT-3' | 91 |
| | R 5'-TCATCTGCGGCTGGGTTTGGTATTTCTTC-3' | |
| **gp130** | F 5'- CATAGTCGTGCCTGTGTGCT-3' | 92 |
| | R 5'-GCCGTCCGAGTACATTTGAT-3' | |
| **SOD1** | F 5'-GAGACCTGGGCAATGTGACT-3' | 89 |
| | R 5'-GTTTACTGCGCAATCCCAAT-3' | |
| **VEGF** | F 5'-TCTACCTCCACCATGCCAAGT-3' | 95 |
| | R 5'-GCTGCGCTGATAGACATCCA-3' | |
| **Endothelin-1** | F 5'-TTCCCGTGATCTTCTCTCTGCT-3' | 92 |
| | R 5'-TCTGCTTGGCAGAAATTCCA-3' | |
| **IL11** | F 5'-GTTTACAGCTCTTGATGTCTC-3' | 94 |
| | R 5'-GAGTCTTTAACAACAGCAGG-3' | |
| **IL6** | F 5'-TAGTCCTTCCTACCCCAATTT-3' | 95 |
| | R 5'-TTGGTCCTTAGCCACTCCTTC-3' | |
| **β2-microglobulin** | F 5'-GGTCTTTCTGGTGCTTGTCTCA-3' | 97 |
| | R 5'-GTTCGGCTTCCCATTCTCC-3' | |
